# Antioxidant Function and Application of Plant-Derived Peptides

**DOI:** 10.3390/antiox13101203

**Published:** 2024-10-06

**Authors:** Zhengqing Zhu, Ziwu Xu, Yuhang Li, Yutong Fan, Yingqian Zhou, Kaixin Song, Lei Meng

**Affiliations:** 1 School of Pharmacy, Hunan University of Chinese Medicine, Changsha 410208, China; z1257624363@163.com (Z.Z.); 13933430502@163.com (Y.L.); 13973352931@163.com (Y.Z.); 17830032702@163.com (K.S.); 2 College of Biology, Hunan University, Changsha 410012, China; xuziwu99@gmail.com; 3 School of Biotechnology, Jiangnan University, Wuxi 214122, China; 18333249368@163.com

**Keywords:** antioxidant activity, plant peptides, oxidative stress, mechanism of action, integrated application

## Abstract

With the development of society and the improvement of people’s health consciousness, the demand for antioxidants is increasing. As a natural antioxidant with no toxic side effects, antioxidant peptides are widely used in food, cosmetics, medicine, and other fields because of their strong antioxidant capacity and easy absorption by the human body. Plant-derived antioxidant peptides have attracted more attention than animal-derived antioxidant peptides because plants are more diverse than animals and produce a large number of protein-rich by-products during the processing of their products, which are the main source of antioxidant peptides. In this review, we summarize the source, structure and activity, other biological functions, mechanism of action, and comprehensive applications of plant antioxidant peptides, and look forward to their future development trends, which will provide a reference for further research and development of plant antioxidant peptides.

## 1. Introduction

Reactive oxygen species (ROS) are small molecules derived from oxygen, which are by-products of the normal metabolism of human cells, mainly including hydroxyl free radicals (^•^OH), superoxide anion free radicals (O_2_^•−^), singlet oxygen, and hydrogen peroxide (H_2_O_2_). It has important functions in the organism, such as promoting cell signaling and proliferation and other life processes [1,2,3]. Studies have shown that excess ROS can unbalance the redox state of cells in vivo, leading to oxidative stress in the organism when it exceeds the effective antioxidant response capacity of cells [4]. Oxidative stress can affect cell ingredients, such as lead to impaired protein synthesis, DNA mutations, RNA damage, and lipid peroxidation [5,6], which in turn leads to a range of diseases such as diabetes, cardiovascular disease, cancer, and Parkinson’s disease [7,8,9,10].

In order to avoid the effects caused by oxidative stress, natural or synthetic antioxidants have been widely used in food, medicine, and cosmetics. However, it has been found that some synthetic antioxidants can cause certain damage to the human body [11,12], so the search for safe and effective natural antioxidants has become a hot topic of concern.

**Figure 1:**
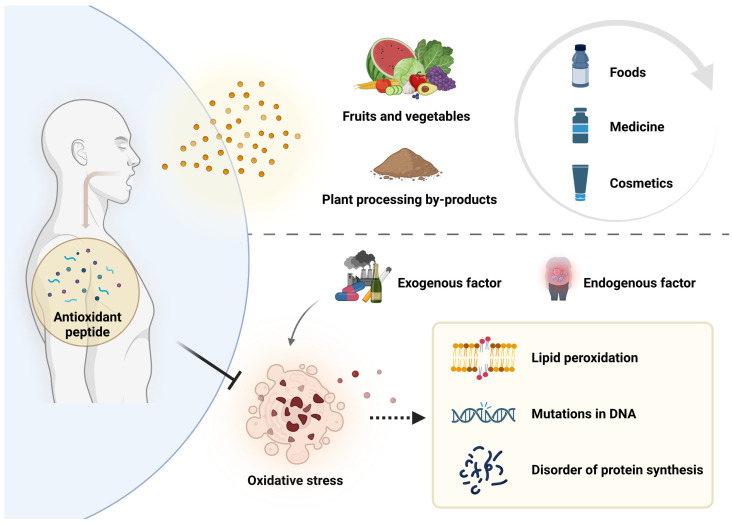
Causes and effects of oxidative stress, and inhibition of oxidative stress by plant antioxidant peptides.

Plant antioxidant peptide is a kind of plant bioactive peptide with antioxidant function, most of which is composed of 2–20 amino acids. Its molecular weight is lower than 0.3 kDa [13] and it can effectively scavenge excess reactive oxygen radicals in the body, protect the normal structure and function of cells and mitochondria, prevent the occurrence of lipid peroxidation, and then play the role of delaying aging and preventing diseases [14,15]. In recent years, antioxidant peptides have attracted wide attention due to their significant antioxidant activity and easy absorption by the human body. Since many antioxidant peptides are derived from plants, it has become a hotspot to extract antioxidant peptides from plants and apply them in the fields of food, medicine, and cosmetics (Figure 1).

This paper summarizes the sources, structures and activities, other biological functions, mechanisms of action, and comprehensive applications of plant antioxidant peptides, discusses the current constraints on the development of the plant antioxidant peptide industry, and looks forward to its future development trends to provide a reference for its further research and development.

## 2. Sources of Plant Antioxidant Peptides

Plant antioxidant peptides have a wide range of sources and exist in all parts of plants. In addition, plant processing is prone to produce a large number of cheap industrial by-products, such as walnut meal and rice bran, which have a high protein content within them and are also regarded as one of the main sources of plant antioxidant peptides. Table 1 divides plant antioxidant peptides according to their sources and lists the amino acid sequences of peptides with antioxidant functions.

## 3. Structure and Activity of Plant Antioxidant Peptides

Studies have shown that the activity strength of antioxidant peptides is related to their structure, length, amino acid composition, and other factors [41,42]. The activity is positively correlated with the hydrophobicity, isoelectric point, and net charge of the peptide and negatively correlated with the molecular weight size [38]. Antioxidant peptides with aromatic or hydrophobic residues are able to exhibit stronger antioxidant properties. The experimental results of antioxidant properties of different antioxidant peptides are listed in Table 2 and their structural features are summarized.

In summary, it was found that most of the plant antioxidant peptides contained high levels of aromatic amino acids and hydrophobic amino acids, which suggests that they have a non-negligible role in the antioxidant function exerted by plant-derived peptides. Yu et al. [37] used the hydrolysate of corn gluten meal to determine its antioxidant activity and found that the peptides with a molecular weight of less than 1 kDa had high antioxidant activity, and three new antioxidant peptides were isolated from them (IEFL, SAADL, and RYLL), which contained the proportion of hydrophobic amino acids of 75%, 60%, and 50%, and the IEFL and RYLL also contained aromatic amino acids. Therefore, it was hypothesized that these high contents of amino acids might be one of the key reasons for the antioxidant function of peptides with molecular weights less than 1 kDa. In addition to the content of the two amino acids mentioned above, other factors such as the content of antioxidant amino acids, the presence of repetitive amino acid sequences, and the position of some amino acids in the peptide chain also have an effect on the level of antioxidant peptide activity in plants. Zhang et al. [22] isolated four peptides, VVFVDRL, VIYVVDLR, IYVVDLR, and IYVFVR, from soybean hydrolyzed protein. The results showed that they were highly active in scavenging free radicals, which might be related to the high content of hydrophobic amino acids and antioxidant amino acids. In addition, the repetitive amino acid sequence “VV” in VVFVDRL, VIYVVDLR, and IYVVDLR may be another reason for the enhanced antioxidant properties.

## 4. Other Biological Functions of Plant Antioxidant Peptides

It has been found that some antioxidant peptides have biological functions such as anticancer, anti-hypertension, and anti-inflammation while exerting antioxidant activities. By synthesizing the literature, other biological functions possessed by some antioxidant peptides were summarized and are presented in Table 3, together with their amino acid sequences and sources.

In conclusion, it was found that the above plant antioxidant peptides were able to exert biological functions other than antioxidant functions by regulating the expression levels of different disease-related factors. To investigate whether four bioactive peptides (corn, wheat, egg white, and soybean) could effectively lower blood pressure, Zou et al. [43] made a solution and injected it into spontaneously hypertensive rats. The results showed that wheat peptide could significantly lower the systolic blood pressure and inhibit the expression levels of angiotensin 2 and tumor necrosis factor-α (TNF-α) in spontaneously hypertensive rats, thus exerting anti-hypertensive function. In addition to anti-hypertension, wheat peptide can significantly reduce the content of MDA in rats by enhancing the activity of antioxidant enzymes, as well as activate the glutathione (GSH) synthesis pathway or provide raw materials for GSH synthesis, and increase the content of GSH in rats, so as to play an antioxidant function. Wei et al. [44] also investigated other biological functions of walnut antioxidant peptides and found that they significantly improved cognitive and memory deficits in bisphenol AF-treated zebrafish and ethanol-treated rats. This result may be related to the ability of walnut antioxidant peptides to promote the expression of neurotrophic factors, such as brain-derived neurotrophic factor (BDNF) and glial-derived neurotrophic factor (GDNF) as well as inhibit oxidative stress.

**Table 3:** Other biological functions of plant antioxidant peptides.

Amino Acid Sequence	Source	Other Biological Functions	Reference
——	*Cicer arietinum* seed	Hypoglycemic	[45]
——	*Amaranthus hypochondriacus* seed	Anti-thrombotic	[46]
——	*Dioscorea polystachya* stem	Anti-hypertensive	[47]
——	*Oryza sativa* by-product	Anti-hypertensive	[48,49]
——	*Triticum aestivum* by-product	Anti-hypertensive	[50]
IF	*Solanum tuberosum* stem	Anti-hypertensive	[19,51]
PWLNFK FSIAWPR GSHWPFGGK	*Chenopodium quinoa* seed	Anti-hypertensive	[52]
PADVTPEEKPEV	*Helianthus annuus* seed	Anti-inflammatory	[32]
——	*Glycine max* seed	Anti-aging Anti-hypertensive Anti-inflammatory	[50,53]
LY GHS RALP	*Brassica rapa* seed	Anti-hypertensive Anti-inflammatory	[28]
QGRPWG PSRADIY AYNIPVNIAR CTLEW VQTL LGYEN GGW VYY LLPF	*Juglans regia* by-product	Anticancer Anti-hypertensive Improvement in cognitive and memory disorders	[33,44,54,55]

## 5. Mechanisms of Action of Plant Antioxidant Peptides

It has been demonstrated that antioxidant peptides exert antioxidant activity mainly through three mechanisms, including direct scavenging of free radicals, chelation of pro-oxidant metal ions, and enhancement of antioxidant defenses, thereby inhibiting oxidative stress in organisms [19,56,57].

### 5.1. Direct Scavenging of Free Radicals

Antioxidant peptides can directly scavenge free radicals by providing hydrogen atoms or electrons, while the hydrogen- or electron-donating effect depends on their amino acid residues [58]. It has been shown that Tyr, Phenylalanine (Phe), and Tryptophan (Trp) can provide electrons to free radicals to transform them into stable molecules while maintaining their structural stability through resonance structures [41,59]. Hydrophobic amino acids such as Leu, Val, Isoleucine (Ile), and Proline (Pro) transfer electrons to free radicals and thus exhibit excellent free radical scavenging activity [60]. The Glutamate (Glu) side chain produced after partial deamidation of Glutamine (Gln) residues can act as a reducing agent to provide electrons to free radicals for scavenging [61]. In addition, the sulfhydryl group on Cys residues not only has the ability to provide electrons but also transfers hydrogen protons to free radicals [62]. This suggests that some amino acid residues can act as both electron and hydrogen donors.

### 5.2. Chelation of Pro-Oxidant Metal Ions

It has been shown that antioxidant peptides can chelate with pro-oxidant metal ions, change the chemical reactivity of the metal, form insoluble metal complexes, or spatially impede metal–lipid interactions, thereby preventing the formation of ROS for antioxidant effects [58]. Positively charged transition metal ions such as Cu^2+^ and Fe^2+^ can form ionic bonds with the carboxyl groups on antioxidant peptides and form coordination bonds with amine functional groups on them [61]. In addition, some groups on the side chains of amino acid residues, such as sulfhydryl groups on Cys, imidazolyl groups on His, indolyl groups on Trp, and hydroxyl groups on Threonine (Thr), can also bind to metal ions and thus exert antioxidant activity [63].

Torres-Fuentes et al. [64] purified metal ion chelating peptides from chickpea protein hydrolysate and investigated their properties. The results showed that these chelating peptides, when combined with pro-oxidant metal ions, contributed to inhibiting ROS production and thus exerted antioxidant effects. In addition, this study also found that the metal chelating activity of chickpea protein hydrolysate was directly proportional to the content of His. Wang et al. [35] purified and identified the antioxidant peptides from cottonseed protein and found that cottonseed protein hydrolysate had good Fe^2+^ chelating activity, and its free radical activity was positively correlated with Fe^2+^ chelating activity. In addition, amino acid composition analysis showed that cottonseed protein hydrolysate was rich in Glu and Aspartic acid (Asp), which could act as an effective metal ion chelator to prevent the formation of free radicals, thus helping the antioxidant peptides to play a role.

### 5.3. Enhancement of the Antioxidant Defence System

Studies have shown that the body’s antioxidant defense system is divided into a non-enzymatic antioxidant system and an enzymatic antioxidant system. The non-enzymatic antioxidant system mainly includes GSH, carotene, melatonin, etc., while the enzymatic antioxidant system mainly includes SOD, CAT, and GPH-Px [58,65].

Since antioxidant peptides can scavenge excess ROS present in the body, they can act as exogenous antioxidants to offset the depletion of endogenous antioxidants in the body. Studies have shown that gamma-glutamyltransferase (GTT) can degrade exogenous and endogenous GSH when stimulated by external conditions such as ethanol, UV light, and toxins [66]. Chen et al. [67] investigated an antioxidant peptide (NDAEYGICGF) purified from microalgae protein hydrolysate and found that it could exhibit antioxidant effects on ethanol-induced oxidative stress in human hepatocellular carcinoma cells (HepG2) by inhibiting the expression level of GTT protein. In addition, the docking model of NDAEYGICGF with GGT suggests that the peptide may be mediated by hydrogen bonds and thus interact with the GGT active site, causing inhibition of its enzymatic activity.

**Figure 2:**
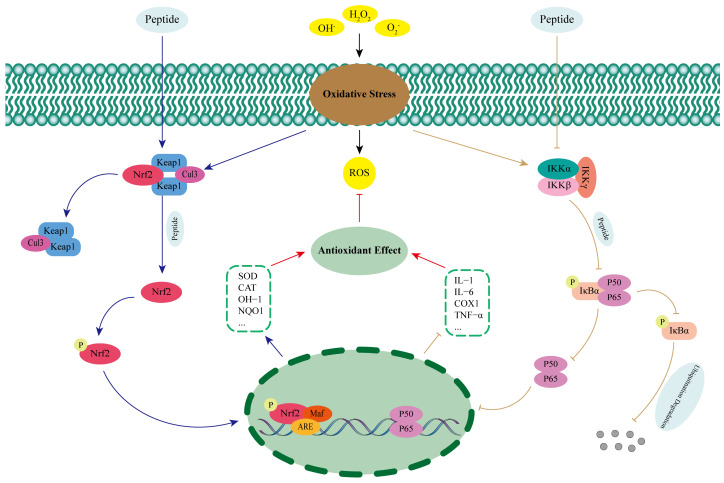
Regulation of antioxidant peptides on the Keap1-Nrf2-ARE and NF-κB signaling pathways.

The nuclear factor-erythroid 2-related factor 2 (Nrf2) is a key factor in the regulation of oxidative stress in humans [68]. Normally, Nrf2 excess in the cytoplasm is mediated by Kelch-like ECH-associated protein 1 (Keap1) binding to cullin-RING E3 ligases 3 (Cul3), which is then degraded [69]. When under oxidative stress, excess ROS promotes the release of Nrf2 from the Nrf2-Keap1-Cul3 complex and into the nucleus, where it forms a heterodimer with V-Maf musculoaponeurotic fibrosarcoma (Maf) and recognizes the antioxidant reaction element (ARE) and binds to it, which in turn promotes the expression of antioxidant enzymes and phase II detoxification enzymes and exerts antioxidant effects (Figure 2) [70,71]. Studies have shown that antioxidant peptides can activate the Keap1-Nrf2-ARE signaling pathway by competitively binding to the active site of Keap1, interfering with the interaction between Keap1 and Nrf2, and promoting the release of Nrf2 and its entry into the nucleus [72]. Wu et al. [73] successfully identified two kinds of antioxidant peptides (IAY and TIL) that could significantly reduce oxidative damage in human umbilical vein endothelial cells (HUVECs) from Bangia fusco-purpurea and explored their antioxidant mechanisms in depth. The results showed that the two peptides were able to significantly increase the expression levels of Nrf2 protein and mRNA, promote Nrf2 nuclear translocation, and activate the expression of antioxidant enzymes and cytoprotective proteins under H_2_O_2_ stimulation. When Nrf2 was silenced, the antioxidant and anti-apoptotic effects of IAY and TIL on H_2_O_2_-induced HUVECs were inhibited. Therefore, it was hypothesized that the two antioxidant peptides could enter the cytoplasm of H_2_O_2_-induced HUVECs and exert antioxidant effects by occupying the binding site between Nrf2 and Keap1. Antioxidant peptides isolated from broken rice and rice bran proteins have also been shown to occupy the binding site between Keap1 and Nrf2 by techniques such as molecular docking and Western Blotting, promoting Nrf2 nuclear translocation, recognizing the ARE, and increasing the expression level of antioxidant enzymes, such as SOD and CAT, thus exerting antioxidant effects and reducing the level of oxidative damage to the cells [68,74].

Nuclear transcription factor-kappa B (NF-κB) is a key intracellular regulatory molecule involved in a variety of physiological processes such as inflammation, innate immunity, and apoptosis, and its most predominantly activated form is a heterodimer consisting of p65 and p50 [75,76]. Under normal physiological conditions, NF−kappa−B inhibitor alpha (IκBα) can bind to NF-κB, mask nuclear localization signals, and inhibit its nuclear translocation. When stimulated by oxidative stress, inhibitor of kappa B kinase (IKK), consisting of three subunits, inhibitor of kappa B kinase alpha (IKKα), inhibitor of kappa B kinase betta (IKKβ), and inhibitor of kappa B kinase gamma (IKKγ), is activated, leading to phosphorylation of IκBα as well as ubiquitination degradation, which in turn promotes NF-κB release. Free NF-κB rapidly translocates to the nucleus and specifically induces the transcription and expression of pro-inflammatory and apoptotic genes, leading to cellular damage (Figure 2) [76,77,78]. Zhang et al. [79] investigated the mechanism of action of antioxidant peptide (YWDHNNPQIR) extracted from rapeseed to alleviate renal fibrosis in diabetic nephropathy and found that it was able to significantly inhibit the expression of p65 in diabetic mice, and at the same time, it had a significant inhibitory effect on the high glucose-induced phosphorylation of p65 and transforming growth factor beta 1 (TGF-β1) in the glomerular thylakoid membrane cells of rats. This suggests that the antioxidant peptide can exert antioxidant effects by inhibiting the NF-κB signaling pathway. Tong et al. [80] investigated the antioxidant mechanism of rice-derived peptide AAGALPS and found that it significantly improved inflammation and oxidative damage in vascular endothelial cells induced by Tumor Necrosis Factor Alpha (TNF-α). Meanwhile, protein immunoblotting experiments revealed that compared with TNF-α alone, the expression level of IKKα was significantly inhibited by the combined treatment of AAGALPS and TNF-α, and the intracellular levels of IκBα were increased. This demonstrated that the antioxidant peptide was able to inhibit the NF-κB pathway and exert antioxidant effects by hindering IKK activation and slowing down IκBα degradation.

## 6. Integrated Application of Plant Antioxidant Peptides (Figure 3)

### 6.1. Food Field

#### 6.1.1. Used as an Antioxidant

It has been found that lipids and other nutrients within food products are susceptible to damage by oxidation reactions during transportation and storage, resulting in changes in color and flavor and even the production of harmful substances [81].

**Figure 3:**
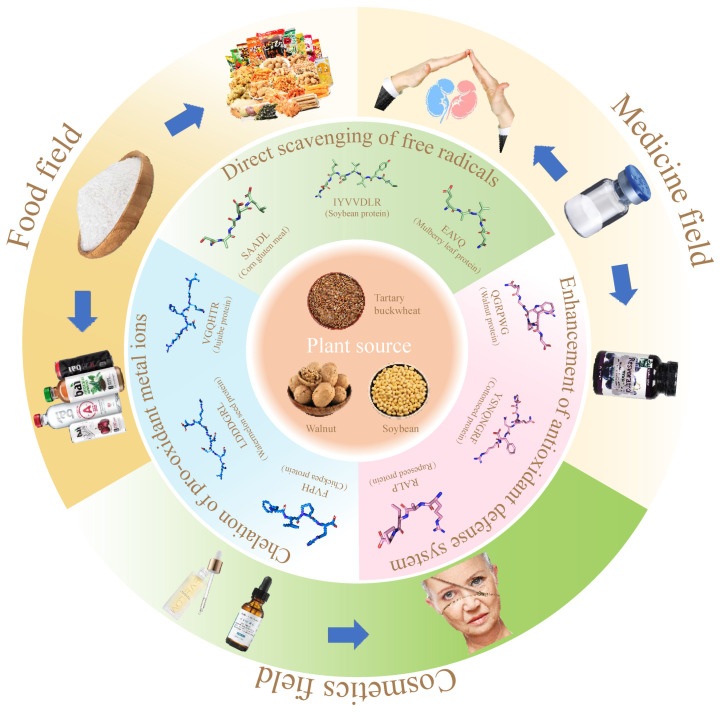
The mechanism of antioxidant peptides from plants and their comprehensive applications in food, medicine, and cosmetics.

To solve this problem, many synthetic antioxidants such as butyl hydroxyisol, butyl hydroxytoluene, or tert-butylhydroquinone are used in foods. Although they are effective in inhibiting the oxidation process of nutrients such as lipids, their safety is still not guaranteed [82]. It has been reported that plant antioxidant peptides have a significant inhibitory effect on lipid peroxidation during food transport and storage, can effectively maintain the stability of food flavor and nutrients, and have higher antioxidant activity compared with synthetic antioxidants [83,84]. Therefore, the use of plant antioxidant peptides as antioxidants and their incorporation into foods can be helpful in alleviating concerns about food safety.

Hu et al. [85] added the hydrolysate of corn gluten meal to pork meal and determined the degree of inhibition of lipid oxidation of pork meal during the 16-day storage period. The results showed that the hydrolysate of corn gluten meal could effectively inhibit lipid peroxidation of pork meal, so it can be added into food as an antioxidant to improve the stability of the product in transport and storage and prolong the shelf life of food.

#### 6.1.2. Development of Functional Products

It has been reported that plant antioxidant peptides are easily absorbed by the intestinal tract and can maintain a relatively intact structure to enter the circulatory system and give full play to their antioxidant functions, so they can be used as a strong candidate for the development of functional foods [86].

Chen et al. [87] isolated three antioxidant peptides, HGEPGQQQR, VAPFPEVFGK, and HNVADPQR, from walnut hydrolyzed protein, which inhibited oxidative stress by acting at key sites in the Keap1-Nrf2 pathway and could significantly improve the survival of ethanol-induced injury in HepG2 cells. Overall, these three antioxidant peptides may serve as potential hepatoprotective agents and be used in the development of functional foods. Samaei et al. [88] explored the functional properties of faba bean protein hydrolysate and investigated its effect on apple juice quality. It was found that the solubility of the broad bean protein hydrolysate was significantly increased, and its foamy ability was also improved to varying degrees. In addition, its hydrolysis products contained a variety of antioxidant peptides, which exerted their antioxidant effects through direct scavenging of free radicals or chelation with metal ions. The apple juice quality effect experiment found that the antioxidant peptide concentration below 3% had no significant effect on the flavor of apple juice except for some bitter taste. It has been found that orange juice can be used as a potential carrier of bioactive peptides [89], which can play a role in the development of functional beverages. Therefore, similar to orange juice, faba bean protein hydrolysate can also be added to apple juice to act as an antioxidant peptide carrier, and thus develop functional drinks with antioxidant effects.

In addition to the above plant protein hydrolysates, other plant protein hydrolysates can be used as functional ingredients and to develop food products with specific functions. For example, cardamom protein hydrolysate not only has excellent antioxidant properties but also has anti-fatigue activity, which can prolong weight-bearing swimming time in mice [89]; moreover, wheat protein and soybean protein hydrolysate can reduce blood pressure in spontaneously hypertensive rats while exerting antioxidant function [50].

### 6.2. Medicine Field

It has been found that oxidative stress caused by excessive ROS leads to many chronic diseases, such as neurological diseases and inflammatory diseases [90]. Antioxidant peptides can effectively remove excess ROS from the body and protect the normal structure and function of cells, so they have become a research hotspot in the medical field.

Diabetic nephropathy (DN) is a severe complication of diabetes mellitus and a major cause of end-stage renal disease [91]. Previous studies have proved that the development of DN is associated with excessive accumulation of extracellular matrix components and oxidative stress [92]. Zhang et al. [79] utilized an antioxidant peptide from rapeseed (YWDHNNPQIR) [93], which has been validated to have antioxidative stress properties, to investigate its pharmacological effects on DN renal fibrosis as well as high-glucose-induced glomerular mesangial dysfunction. The results showed that YWDHNNPQIR could inhibit DN renal fibrosis by inhibiting the MAPK and NF-κB pathways, and it is expected to be designed as a natural drug for treating DN renal fibrosis.

In addition to rapeseed antioxidant peptides, other plant antioxidant peptides have also been shown to have disease-delaying and therapeutic effects. For example, the antioxidant peptide (RQSHFANAQP) extracted from chickpeas can effectively inhibit the proliferation of breast cancer cells by increasing the p53 content [94]. Three peptide fractions (>10 kDa, 5–10 kDa, and <5 kDa) obtained from germinated soybeans have good antioxidant activity and can effectively inhibit the proliferation of breast and cervical cancer cell lines [95]. In addition, bioactive peptides (PELF and IALLIPF) isolated from foxtail millet can also inhibit the production of intracellular ROS and MDA, thus showing antioxidant activity, and it can alleviate inflammation by inhibiting the production of nitric oxide and pro-inflammatory cytokines [96].

Zhao et al. [97] identified five kinds of antioxidant peptides by ABTS after hydrolyzing wheat protein and treating them in an environment simulating the gastrointestinal enzyme system. It found that three peptides, CGFPGHC, RNF, and WF, lost their antioxidant properties. Therefore, preventing antioxidant peptides from being degraded is an issue that must be emphasized during drug preparation. Arturo et al. [98] used ionic gel and spray freeze-drying technology to embed antioxidant peptides to prepare gel nanoparticles. They found that antioxidant peptide activity could be well maintained and had little effect on cell viability. This suggests that nanoparticles can be used to embed antioxidant peptides and design an antioxidant peptide nanoparticle for delivery to the colon, which helps to maintain the bioavailability and pharmacological activity of antioxidant peptides.

### 6.3. Cosmetics Field

Modern studies have shown that skin aging is a process of degenerative changes in the appearance of the skin and the structure and function of the skin layers caused by intrinsic and extrinsic factors [99]. With the aging of the population in today’s society and the continuous improvement of living standards, the demand for anti-skin aging products is increasing, and how to prevent and delay skin aging has become a hot spot of concern.

Zhu et al. [100] isolated seven antioxidant peptides, including YLSF, LPSYVN, and SPHWNVN, from apricot using papain and the results of antioxidant activity assay showed that the scavenging rate of the above antioxidant peptides on ABTS free radicals was higher than that of GSH and ascorbic acid. In addition, the study also found that the above antioxidant peptides were able to significantly inhibit the extent of UV damage in mice. And based on the 4D label-free quantitative proteomics results, it was speculated that the apricot antioxidant peptides might inhibit UV-induced oxidative damage through three key pathways, including the base excision repair pathway. Mo et al. [101] used Lactobacillus plantarum to forage rice, extracted and purified a short peptide mixture (RFP) with less than 11 amino acids from the fermentation broth, and evaluated the antioxidant ability of RFP and its ability to remove ROS and MDA in human skin fibroblasts under oxidative stress induced by UVA. The results showed that RFP inhibited and delayed skin aging by promoting Nrf2 nuclear translocation, scavenging lipid oxidation products and excessive ROS, and enhancing the expression of antioxidant enzyme genes downstream of the Keap1-Nrf2-ARE pathway. Therefore, plant antioxidant peptides can be used as one of the new sources of raw materials for anti-aging cosmetics.

## 7. Conclusions

In recent years, as people have become increasingly aware of the important role of redox state balance in health status and the maintenance of health status, the production of antioxidant products that are safe, non-toxic, and have excellent antioxidant properties has become one of the demands of people. Plant-derived antioxidant peptides have attracted much attention in recent years, and have been used for product development in the food, medicine, and cosmetic fields because of their safety, easy absorption by the human body, excellent antioxidant properties, and wide range of sources. This paper summarizes the sources, structures, activities, and other biological functions of plant antioxidant peptides, and also elaborates on the mechanism of plant antioxidant peptides, detailing the antioxidant function of plant antioxidant peptides through Keap1-Nrf2-ARE, NF-κB, and other pathways. In addition, the application of plant antioxidant peptides in food, medicine, and cosmetics is also introduced.

Although the sources of plant antioxidant peptides are very wide, their extraction sources are mainly from various organs of plants, such as flowers, fruits, and seeds. It is less common to extract antioxidant peptides from the by-products of plant processing products. Numerous studies have shown that the by-products of processed plant products are high in protein content and yield, but are often discarded directly or used as low-value waste or feed, which seriously hampers the economic development of this plant industry [37,102]. In addition, the industrial-scale production of plant antioxidant peptides is also a major problem that needs to be solved urgently. The current method of producing plant antioxidant peptides is still relatively small-scale, time-consuming, and expensive, and the development of the plant antioxidant peptide industry is severely limited due to the high cost of large-scale production and subsequent purification of plant antioxidant peptides.

Therefore, in order to further develop plant antioxidant peptides in the food, medicine, and cosmetic fields, research on the by-products produced during the processing of plant products is essential. The extraction and purification techniques of plant antioxidant peptides also need to be improved, which is indispensable for their industrial-scale production. It is believed that with the development of molecular biology, cell biology, and bioinformatics, as well as the cross-integration of various disciplines, these problems will be overcome one by one, and the development and application of plant antioxidant peptides will take a new step forward.

## Figures and Tables

**Table 1:** Sources of plant antioxidant peptides.

Amino Acid Sequence	Source	Genus and Species	Reference
VDPYFNK DGGSDYLGK	Flower	*Crocus sativus*	[16]
GWLK VGQHTR	Fruit	*Ziziphus jujuba*	[17]
VTYM	Root and stem	*Zingiber officinale*	[18]
IF	Stem	*Solanum tuberosum*	[19]
SVL RDY EAVQ	Leaf	*Morus alba*	[20]
AH LY TW LW ELT LHK LHV LAH LLPH ALLPH	Seed	*Vigna radiata*	[21]
IYVFVR IYVVDLR VVFVDRL VIYVVDLR	Seed	*Glycine max*	[22]
LDLVKPQ YGRDEISV	Seed	*Cannabis sativa*	[23]
CTGFVAVR LRGENDQR GFIVQAQDLK SFFLAGQSQQGR	Seed	*Fagopyrum tataricum*	[24]
SF QY	Seed	*Moringa oleifera*	[25]
RDPEER KELEEK LDDDGRL DAAGRLQE GFAGDDAPRA	Seed	*Citrullus lanatus*	[26]
FVPH ALEPDHR SAEHGSLH TETWNPNHPEL	Seed	*Cicer arietinum*	[27]
LY GHS RALP	Seed	*Brassica rapa*	[28]
TETWNPNHPEL	Seed	*Vicia faba*	[29]
VGPWQK	Seed	*Artocarpus heterophyllus*	[30]
RDRHQKIG TDRHQKLR SYPTECRMR RENIDKPSRA MNDRVNQGE	Seed	*Sesamum indicum*	[31]
AGEQGFEYVTFR GGVPRSGEQEQQ	Seed	*Sesamum indicum*	[31]
DVAMPVPK VETGVIKPG TTHTNPPPEAE PADVTPEEKPEV LTHPQHQQQGPSTG	Seed	*Helianthus annuus*	[32]
QGRPWG PSRADIY AYNIPVNIAR	By-product	*Juglans regia*	[33]
QFLLAGR ASPKPSSA RGQVIYVL	By-product	*Chenopodium quinoa*	[34]
QWDRQ WDTRGQ YSNQNGRF	By-product	*Gossypium hirsutum*	[35]
FSAP EAAY QEPLLR PVETVR VLRPPLS	By-product	*Paeonia ostii*	[36]
IEFL RYLL SAADL	By-product	*Zea mays*	[37]
WAF GGIF AWFS YLLLK YGIKVGYAIP LPWRPATNVF	By-product	*Elaeis guineensis*	[38]
LLLRW GDMNP	By-product	*Oryza sativa*	[39]
EEGER GMEEER	By-product	*Cocos nucifera*	[40]

**Table 2:** Structural characteristics and activities of plant antioxidant peptides.

Amino Acid Sequence	Source	Experimental Result	Structural Feature	Reference
VDPYFNK DGGSDYLGK	*Crocus sativus* flower	Scavenging capacity for ABTS free radicals (IC_50_): 0.1657 mg/mL VDPYFNK; 0.2930 mg/mL DGGSDYLGK. Scavenging capacity for DPPH free radicals (IC_50_): 0.6411 mg/mL VDPYFNK; 0.3901 mg/mL DGGSDYLGK. Reducing capacity for Fe^3+^: 0.167 µmol/mL~0.063 µmol/mL VDPYFNK; 0.144 µmol/mL~0.0549 µmol/mL DGGSDYLGK. Effects on HepG2 oxidative stress cell model: enhancement of SOD, CAT, and other antioxidant enzyme activities and reduction in intracellular MDA content.	Contains hydrophobic and aromatic amino acids.	[16]
GWLK VGQHTR	*Zizyphus jujuba fruit*	Scavenging capacity for DPPH free radicals (EC_50_): 0.5 ± 0.051 mg/mL GWLK; 0.6 ± 0.065 mg/mL VGQHTR. Chelating capacity with Fe^2+^ (EC_50_): 0.86 mg/mL GWLK; 1.18 mg/mL VGQHTR.	Rich in hydrophobic, aromatic, and basic amino acids.	[17]
VTYM	*Zingiber officinale root and stem*	Scavenging capacity for ABTS free radicals (EC_50_): 19.9 ± 2.1 μmol/L. Scavenging capacity for DPPH free radicals (EC_50_): 24.0 ± 3.7 μmol/L.	Contains hydrophobic and aromatic amino acids.	[18]
SVL RDY EAVQ	*Morus alba* leaf	Effect on cellular antioxidant activity (CAA): 1706 μM QE/100 g SVL; 2204 μM QE/100 g RDY; 1501 μM QE/100 g EAVQ.	Contains hydrophobic amino acids and the presence of Tyr at the C-terminus of the peptide chain.	[20]
AH LY TW LW ELT LHK LHV LAH LLPH ALLPH	*Vigna radiata* seed	Scavenging capacity of mung bean hydrolyzed protein for ABTS free radicals (IC_50_): 18.06 ± 0.18 μg/mL. Scavenging capacity of mung bean hydrolyzed protein for DPPH free radicals (IC_50_): 11.10 ± 0.02 μg/mL. Chelating ability of mung bean hydrolyzed protein with Fe^2+^ (IC_50_): 3.78 ± 0.59 μg/mL.	Rich in hydrophobic amino acids and His.	[21]
IYVFVR IYVVDLR VVFVDRL VIYVVDLR	*Glycine max* seed	Scavenging capacity for ABTS free radicals: 3.22 ± 0.1 mM TE/mg IYVFVR; 3.72 ± 0.3 mM TE/mg IYVVDLR; 3.14 ± 0.1 mM TE/mg VVFVDRL; 3.34 ± 0.2 mM TE/mg VIYVVDLR. Scavenging capacity for DPPH free radicals: 14.4 ± 0.6 μM TE/mg IYVFVR; 17.8 ± 0.9 μM TE/mg IYVVDLR; 14.9 ± 0.7 μM TE/mg VVFVDRL; 16.1 ± 0.5 μM TE/mg VIYVVDLR. Reducing capacity for Fe^3+^: 62.9 ± 0.5 mM Fe^2+^/mg IYVFVR; 68.9 ± 1.4 mM Fe^2+^/mg IYVVDLR; 63.1 ± 1.2 mM Fe^2+^/mg VVFVDRL; 53.4 ± 1.2 mM Fe^2+^/mg VIYVVDLR. Absorption capacity for oxygen free radicals: 136 ± 3.8 μM TE/mg IYVFVR; 139 ± 1.0 μM TE/mg IYVVDLR; 136 ± 1.9 μM TE/mg VVFVDRL; 140 ± 2.0 μM TE/mg VIYVVDLR.	Rich in hydrophobic and antioxidant amino acids; the presence of repeated amino acids in the antioxidant peptide sequence.	[22]
LDLVKPQ YGRDEISV	*Cannabis sativa* seed	Clearance rate of hemp seed hydrolyzed protein for ABTS free radicals: 52.3 ± 0.1%. Chelation rate of hemp seed hydrolyzed protein for Fe^2+^: 52.9 ± 0.9%. Clearance rate of hemp seed hydrolyzed protein for hydroxyl radical: 50.9 ± 1.3%. Effects of hemp seed hydrolyzed protein on HepG2 oxidative stress cell model: enhancement of SOD, CAT, and GSH-Px and other antioxidant enzyme activities.	Lower molecular weight; contains hydrophobic and aromatic amino acids; Val is present at the C-terminus of the peptide chain; Tyr is present at the N- or C-terminus of the peptide chain.	[23]
CTGFVAVR LRGENDQR GFIVQAQDLK SFFLAGQSQQGR	*Fagopyrum tataricum* seed	Effects of tartary buckwheat hydrolyzed protein on Caco-2 oxidative stress cell model: enhancement of SOD, CAT, and other antioxidant enzyme activities and reduction in intracellular MDA content.	Val is present at the C-terminus of the peptide chain; higher hydrophilic amino acid content; Cys is present at the N-terminus of the peptide chain.	[24]
SF QY	*Moringa oleifera* seed	Scavenging capacity for ABTS free radicals (EC_50_): 0.32 ± 0.022 mg/mL SF; 0.33 ± 0.041 mg/mL QY. Scavenging capacity for DPPH free radicals (EC_50_): 1.37 ± 0.086 mg/mL SF; 0.75 ± 0.065 mg/mL QY. Effects on Chang liver oxidative stress cell model: enhancement of SOD, CAT, and other antioxidant enzyme activity and reduction in intracellular ALT, AST, and MDA content.	Shorter peptide chains; contains hydrophobic and aromatic amino acids.	[25]
RDPEER KELEEK LDDDGRL DAAGRLQE GFAGDDAPRA	*Citrullus lanatus* seed	Scavenging capacity for ABTS free radicals (IC_50_): 0.54 ± 0.02~1.23 ± 0.03 mg/mL. Scavenging capacity for DPPH free radicals (IC_50_): 0.216 ± 0.01~0.435 ± 0.03 mg/mL. Absorption capacity for oxidative free radicals: 82.36 ± 1.2~130.67 ± 2.2 μM TE/mg. Effects on HepG2 oxidative stress cell model: enhancement of SOD, CAT, and GSH-Px and other antioxidant enzyme activities and reduction in intracellular MDA content.	Lower molecular weight; contains acidic, hydrophobic, and antioxidant amino acids; the presence of repeated amino acids in the antioxidant peptide sequence; Leu is present at the N-terminus of the peptide chain.	[26]
FVPH ALEPDHR SAEHGSLH TETWNPNHPEL	*Cicer arietinum* seed	——	Rich in acidic, basic, and hydrophobic amino acids; hydrophobic amino acids are present at the N- or C-terminus of the peptide chain; His and Arg are present at the C-terminus of the peptide chain.	[27]
VGPWQK	*Artocarpus heterophyllus* seed	Scavenging capacity for ABTS free radicals (EC_50_): 1.00 ± 0.00 mg/mL.	Contains hydrophobic amino acids and is present at the N-terminal and C-terminal ends of the peptide chain.	[30]
RDRHQKIG TDRHQKLR SYPTECRMR RENIDKPSRA MNDRVNQGE AGEQGFEYVTFR GGVPRSGEQEQQ	*Sesamum indicum* seed	Scavenging capacity for ABTS free radicals (IC_50_): 6.414 ± 0.292 mg/mL RDRHQKIG; 1.145 ± 0.042 mg/mL TDRHQKLR;10.004 ± 0.000 mg/mL SYPTECRMR; 2.842 ± 0.073 mg/mL RENIDKPSRA; 7.390 ± 0.387 mg/mL MNDRVNQGE; 3.958 ± 0.036 mg/mL AGEQGFEYVTFR;10.720 ± 0.047 mg/mL GGVPRSGEQEQQ. Scavenging capacity for DPPH free radicals (IC_50_): 4.648 ± 0.021 mg/mL RDRHQKIG; 6.353 ± 0.035 mg/mL TDRHQKLR; 10.105 ± 0.018 mg/mL SYPTECRMR; 23.650 ± 0.117 mg/mL RENIDKPSRA; 6.763 ± 0.084 mg/mL MNDRVNQGE; 1.141 ± 0.012 mg/mL AGEQGFEYVTFR; 10.601 ± 0.023 mg/mL GGVPRSGEQEQQ.	Contains Cys, Met, and aromatic amino acids; larger amino acids at the C-terminal end of the peptide chain; the presence of electrostatic interaction.	[31]
QGRPWG PSRADIY AYNIPVNIAR	*Juglans regia* by-product	Scavenging capacity of walnut meal hydrolyzed protein for DPPH free radicals (IC_50_): 674.2 μg/mL. Absorption capacity of walnut meal hydrolyzed protein for oxygen free radicals: 591.4 μmol TE/g.	Rich in hydrophobic amino acids; the PWG sequence is present at positions 4–6 of the peptide chain.	[33]
QFLLAGR ASPKPSSA RGQVIYVL	*Chenopodium quinoa* by-product	Clearance rate of quinoa bran hydrolyzed protein for ABTS free radicals: 58.29~74.28%. Chelation rate of quinoa bran hydrolyzed protein for Fe^2+^: 32.54~82.48%. Scavenging capacity of quinoa bran hydrolyzed protein for hydroxyl radicals: 61.69~117.46 µM.	Shorter peptide chains; contains hydrophobic and aromatic amino acids, with hydrophobic amino acids predominating.	[34]
QWDRQ WDTRGQ YSNQNGRF	*Gossypium hirsutum* by-product	Scavenging capacity of cottonseed meal hydrolyzed protein for ABST free radicals (EC_50_): 2.05 ± 0.02 mg/mL. Scavenging capacity of cottonseed meal hydrolyzed protein for DPPH free radicals (EC_50_): 0.49 ± 0.02 mg/mL. Chelating capacity of cottonseed meal for Fe^2+^ (EC_50_): 0.99 ± 0.03 mg/mL. Scavenging capacity of cottonseed meal hydrolyzed protein for hydroxyl radicals (EC_50_): 2.21 ± 0.12 mg/mL.	Rich in acidic/alkaline, hydrophobic, and aromatic amino acids.	[35]
FSAP EAAY QEPLLR PVETVR VLRPPLS	*Paeonia ostii* by-product	Clearance rate for ABTS free radicals: 98.5% ± 1.1% EAAY. Clearance rate for hydroxyl radicals: 61.9% ± 1.3% EAAY.	Contains hydrophobic and aromatic amino acids; the presence of repeated amino acids in the antioxidant peptide sequence; Ala and Tyr are present at the c-terminus of the peptide chain.	[36]
IEFL RYLL SAADL	*Zea mays* by-product	Scavenging capacity for ABTS free radicals (IC_50_): 0.122 mg/mL RYLL. Scavenging capacity for DPPH free radicals (EC_50_): 0.180 mg/mL RYLL.	Lower molecular weight; contains hydrophobic and aromatic amino acids, with hydrophobic amino acids predominating; the presence of repeated amino acids in the antioxidant peptide sequence.	[37]
WAF GGIF AWFS YLLLK YGIKVGYAIP LPWRPATNVF	*Elaeis guineensis* by-product	Scavenging capacity for DPPH free radicals (IC_50_): 1.310 μM WAF; 0.350 μM GGIF; 1.360 μM AWFS; 0.948 μM YLLLK; 1.090 μM YGIKVGYAIP; 1.070 μM LPWRPATNVF. Chelating capacity with Fe^2+^ (IC_50_): 2.363 μM WAF;1.498 μM GGIF; 0.002 μM AWFS; 0.218 μM YLLLK;0.087 μM YGIKVGYAIP; 0.001 μM LPWRPATNVF.	Lower molecular weight; contains hydrophobic and aromatic amino acids.	[38]
LLLRW GDMNP	*Oryza sativa* by-product	Scavenging capacity for DPPH free radicals (IC_50_): 0.131 ± 0.008 mg/mL LLLRW; 0.120 ± 0.007 mg/mL GDMNP. Scavenging capacity for superoxide anion radicals (IC_50_): 0.430 ± 0.012 mg/mL LLLRW; 0.400 ± 0.008 mg/mL GDMNP. Scavenging capacity for hydroxyl radicals (IC_50_): 0.380 ± 0.012 mg/mL LLLRW; 0.370 ± 0.009 mg/mL GDMNP. Chelating capacity with Fe^2+^ (IC_50_): 0.024 ± 0.008 mg/mL LLLRW; 0.068 ± 0.010 mg/mL GDMNP.	Rich in hydrophobic and aromatic amino acids.	[39]
EEGER GMEEER	*Cocos nucifera* by-product	Scavenging capacity for ABTS free radicals (IC50): 6.54 ± 0.14 mmol/L EEGER; 0.46 ± 0.067 mmol/L GMEEER. Scavenging capacity for DPPH free radicals (IC50): 11.06 ± 0.18 mmol/L EEGER; 6.98 ± 0.29 mmol/L GMEEER.	Contains hydrophobic amino acids; Arg is present at the C-terminus of the peptide chain.	[40]

SOD: superoxide dismutase, CAT: catalase, GSH-Px: glutathione peroxidase, ALT: alanine aminotransferase, AST: aspartate aminotransferase, MDA: malondialdehyde, Tyr: tyrosine, His: histidine, Val: valine, Cys: cysteine, Leu: leucine, Arg: arginine, Met: methionine, and Ala: alanine.
